# The mediating effect of health anxiety in the relationship between functional somatic symptoms and illness behavior in Chinese inpatients with depression

**DOI:** 10.1186/s12888-019-2246-9

**Published:** 2019-08-27

**Authors:** Yue-Jiao Ma, Dong-Fang Wang, Ming Yuan, Jiang Long, Shu-Bao Chen, Qiu-Xia Wu, Xu-Yi Wang, Tie-Qiao Liu

**Affiliations:** 1 Department of Psychiatry, The Second Xiangya Hospital, Central South University, The China National Clinical Research Center for Mental Health Disorders, Chinese National Technology Institute of Psychiatry, Key Laboratory of Psychiatry and Mental Health of Hunan Province, No. 139, Middle Renmin Road, Changsha, Hunan 410011 People’s Republic of China; 2 Psychosomatic health institute of the Third Xiangya Hospital, Central South University, Changsha, 410013 Hunan People’s Republic of China; 3 Laboratory for Experimental Psychopathology, Psychological, Science Research Institute, Université Catholique de Louvain, Louvain-la-Neuve, Belgium

**Keywords:** Depression, Functional somatic symptoms, Illness behavior, Health anxiety, Mediating effect

## Abstract

**Background:**

Functional somatic symptoms in depression disorder may cause inappropriate illness behavior hindering the treatment process. Health anxiety may play a role in this relationship, but few studies have examined it. The current study aimed to investigate the role of health anxiety in the relationship between functional somatic symptoms and illness behavior in patients with depression.

**Methods:**

The present study recruited 323 hospitalized patients with depression to complete the Patient Health Questionnaire-15, Whiteley-Index-7, and Scale for the Assessment of Illness Behavior, then constructed a structural equation model to examine whether health anxiety mediated the relationship between functional somatic symptoms and illness behavior.

**Results:**

The results showed significant correlations between any two of the three variables of interest. More importantly, health anxiety played a partially mediating role (42.86%) in the relationship between functional somatic symptoms and illness behavior. Further analysis suggested that elderly patients reached higher health anxiety than younger patients when their functional somatic symptoms were mild.

**Conclusions:**

These results highlight that health anxiety may mediate the influence of functional somatic symptoms on illness behavior. The implications of assessing and intervening in health anxiety in patients with depression were discussed.

## Background

Somatic symptoms (SSs) without physical cause are very common in mental illness, such as depression disorder [1–4]. The proportion of functional SSs (FSSs) can reach up to 80% in patients with depression [5]. Most patients with depression incorrectly attribute FSSs to physical conditions, reporting somatic rather than emotional symptoms in their primary care [6]. Consequently, patients with depression frequently use inappropriate forms of illness behavior (IB)—namely, the actions taken to deal with the health issue—such as frequent doctor visits and asking for more body scanning [7–9], treatment nonadherence and more frequent polypharmacy [10], and higher rates of unemployment [1]. This misdirecting or unnecessary IB can probably result in misdiagnosis, treatment delays, rehabilitation barriers, and huge waste of health care services [8, 11, 12]. Therefore, it would be very helpful to clarify the mechanism of how FSSs affect IB.

Health anxiety (HA) may play an important role in the relationship between FSSs and IB. HA is defined as the extensive worry that people can have about their health [13] with intensity ranging from none to severe; hypochondriasis (in the Diagnostic and Statistical Manual of Mental Disorders, Fourth Edition [DSM-IV]) or illness anxiety disorder (in the DSM-V) is an extreme form [14]. FSSs are strongly positively correlated with HA with evidence that patients with severe FSSs often accompanied by higher HA [15–17] tend to worry about their bodily signs and other ambiguous health-related symptoms [18]. In addition, evidence shows that higher HA can cause more inappropriate IB [16, 19, 20]. Therefore, it is worth examining whether HA functions as an intermediary mechanism between FSSs and IB.

The role HA plays between FSSs and IB may be that of a mediator. Some researchers have proposed that IB in patients with FSSs may depend on the underlying psychological pathology, which is closely related to HA, rather than the FSSs themselves [9]. In addition, previous studies showed that both FSSs and HA can predict IB [1, 9, 16]. FSSs appear to share a common neurologic pathway with depression, which is based on neurotransmitter dysregulation of serotonin and norepinephrine in depression disorder [21]. HA affects functional physical symptoms, but it is not the main factor of FSSs. On the contrary, the degree of FSSs will aggravate the level of HA [15], and HA is more contingent and can be changed, for example, by cognitive-behavioral therapy [18]. Thus, it is reasonable to assume that the affecting direction between FSSs and HA should be from the former to the latter rather than the reverse. Even though the relationships among FSSs, HA, and IB have received attention, few empirical studies have directly tied these variables together to examine the possible intermediary processes.

Accordingly, we propose the main hypothesis of the present study—namely, that the influence of FSSs on IB in patients with depression is probably mediated by their HA. Specifically, severe FSSs may produce higher HA, further causing more abnormal IB. To verify the hypothesis, we recruited a sample of Chinese inpatients with depression, whose non-Western cultural context may be associated with higher rates of somatic presentation than in Western or developed countries [22], and built a structural equation model based on structured measurements. We were also interested in whether patient age would have moderating effects in the mediation model of the above hypothesis as previous studies have shown that age-related differences exist in HA [23], medical utilization [24], and help-seeking behavior [25].

## Methods

### Participants and procedure

In this cross-sectional study, 323 inpatients with depression were recruited using convenient sampling from two large hospitals in China from April 2017 to March 2018: The Second Xiang-Ya Hospital, Central South University, and the Second Affiliated Hospital of Guizhou Medical University. All patients enrolled needed to meet the recruitment criteria: 1) diagnosis of major depressive disorder according to the DSM-V by two psychiatrists with more than five years’ clinical experience and 2) ability to understand the contents of all measures. Exclusion criteria were as follows: 1) comorbidity with bipolar disorder, schizophrenia, alcohol/substance use disorders, and other psychotic disorders and 2) major medical abnormalities, including central nervous system diseases or acute, unstable, or life-threatening medical illnesses (e.g., cancer, infections). All participants provided signed, informed consent to participate in this study. Ethical approval was obtained from the Mental Health Institute of Central South University Second Xiang-Ya Hospital.

Following the completion of informed consent forms, patients filled in a series of questionnaires including the Patient Health Questionnaire (PHQ-15), the scale for the Assessment of Illness Behavior (SAIB), and the Whiteley Index-7 (WI-7). Basic information such as gender, age, education, and illness duration was extracted from the patients’ medical records.

### Measures

#### Functional somatic symptoms

FSSs were assessed with the Chinese version of the PHQ-15, which was translated and revised by Lee et al. [26] from the original version [27]. The questionnaire involves 15 common SSs that account for more than 90% of the symptoms seen in primary care. The patients were asked to rate the severity of their symptoms during the past 4 weeks on a 3-point Likert scale: 0 (“not bothered at all”), 1 (“bothered a little”), or 2 (“bothered a lot”). Thus, the total symptom severity score varied from 0 to 30 with higher scores indicating severer SSs. The Chinese version of the PHQ-15 exhibited satisfactory internal consistency (Cronbach’s alpha = 0.79) [26]. The Cronbach’s alpha in the present study was 0.86.

#### Illness behavior

IB was assessed with the Chinese version of the SAIB [28], which was translated and revised from the original version developed by Rief et al. [29]. The Chinese version of the SAIB is a 23-item self-report questionnaire composed of 5 subscales of diagnosis verification (e.g., concerning my diagnosis, I always ask for a second medical opinion), expression of symptoms (e.g., I often try to explain my current state of health to other people), medication/treatment (e.g., due to my complaints, I have already tried alternative medical treatments), illness consequences (e.g., I am not able to concentrate on my work when suffering from physical complaints), and body scanning (e.g., I pay a lot of attention to the different processes going on within my body). Each item is scored on a 4-point Likert scale that varies from 0 to 3 (0 = “ I agree completely”; 1 = “ I partially agree”; 2 = “ I partially disagree”; 3 = “I disagree completely”). Lower scores represent severer dysfunctional IB. Internal consistency of the Chinese version of the SAIB was found to be 0.88, and Cronbach’s alphas across the 5 subscales were between 0.61 to 0.82. Cronbach’s alpha values in the present study were between 0.53 to 0.83, and 0.88 for the total score on the SAIB.

#### Health anxiety

The Chinese version of the WI-7 [30], which was originally developed by Fink et al. [31], was used to assess HA. This questionnaire consists of 7 items, and each item has a dichotomous choice of “no” or “yes” (0 = “ yes ”; 1 = “no”). The total score (range 0–7) was obtained additively; higher scores indicate higher HA. The Chinese version of the WI-7 exhibited satisfactory internal consistency (Cronbach’s alpha = 0.73) [30]. The Cronbach’s alpha in the present study was 0.81.

### Statistical analyses

Descriptive statistics and Pearson’s correlation analyses of three variables (FSS, HA, and IB) were conducted with SPSS 22.0 software. The mediating role of HA in the relationship between FSSs and IB was tested using structural equation modeling (SEM) in AMOS 21.0 with the maximum likelihood estimation method conducted for model estimation. The goodness of fit of the model was evaluated using the following indices: CMIN/DF (a value between 1 and 5 indicates acceptable fit between hypothetical model and sample data), RMSEA (< 0.1 reflects reasonable model fit) [32], SRMR (< 0.08 indicates acceptable fit) [33], and CFI (> 0.9 indicates acceptable fit) [34]. A multivariate regression analysis of FSS, HA, IB, and age was performed with Hayes’ SPSS-PROCESS program model 59 to examine the adjusted moderating effect model of age [35]. Bootstrapped method was used to estimate the significance of indirect effects by 5000 bootstraps and 95% confidence interval.*P*-values < 0.05 were considered statistically significant.

## Results

### Sample characteristics

A total of 323 inpatients with depression were collected in our study; 153 (47.7%) were female. Age ranged from 14 to 68 years, and the mean ± SD of the total sample was 33.84 ± 12.35 years (34.16 ± 12.91 in male and 33.48 ± 11.72 in female participants). The duration of education was 12.60 ± 3.30 years. The mean duration of illness (according to electronic medical records) was 21.8 ± 47.17 months.

### Correlational analyses

Table 1 shows the results of the correlational analyses. The PHQ-15 score was negatively correlated with the SAIB score (*r* = − 0.50) and its five subscales (*r* = − 0.32~ − 0.47), which means that the severer the SSs, the severer the dysfunctional IB. The PHQ-15 score was positively correlated with the WI-7 score (*r* = 0.53). In addition, WI-7 also negatively correlated with SAIB (*r* = − 0.53) and its five subscales (*r* = − 0.21~ − 0.55), which means that people who have higher HA will have severer dysfunctional IB. The above results support the hypothesis of relationship among the three, and we conducted an intermediary model to further validate the hypothesis.

**Table 1:** Means, standard deviations, and correlations for all variables (*n* = 323)

	mean	SD	1	2	3	4	5	6	7	8
1.SAIB(Total)	51.59	12.08	1							
2.DV	8.34	2.99	0.73^**^	1						
3.ES	12,16	3.40	0.69^**^	0.33^**^	1					
4.MT	13.00	3.95	0.77^**^	0.44^**^	0.44^**^	1				
5.IC	8.00	2.45	0.62^**^	0.40^**^	0.33^**^	0.24^**^	1			
6.S	10.08	3.70	0.81^**^	0.54^**^	0.37^**^	0.52^**^	0.48^**^	1		
7.PHQ-15	12.79	6.06	−0.50^**^	− 0.24^**^	− 0.32^**^	−0.41^**^	− 0.36^**^	−0.47^**^	1	
8.WI-7	5.14	2.07	−0.53^**^	− 0.39^**^	− 0.21^**^	− 0.37^**^	−0.41^**^	− 0.55^**^	0.53^**^	1
9.age	33.84	12.35	−0.17^**^	−0.03	− 0.11	−0.12^**^	− 0.09	−0.17^**^	0.17^**^	020^**^

Note: M—means; SD—standard deviation

WI-7: Whiteley Index-7 (assessing health anxiety); SAIB: the Scale for the Assessment of Illness Behavior (assessing illness behavior); PHQ-15: the Patient Health Questionnaire (assessing somatic symptoms); DV: Diagnosis evaluation; ES: expression symptoms; MT: medication and treatment; IC: Illness consequences; S: Scanning;

**Significant at 0.01 level

### Structural equation modeling

**Fig. 1:**
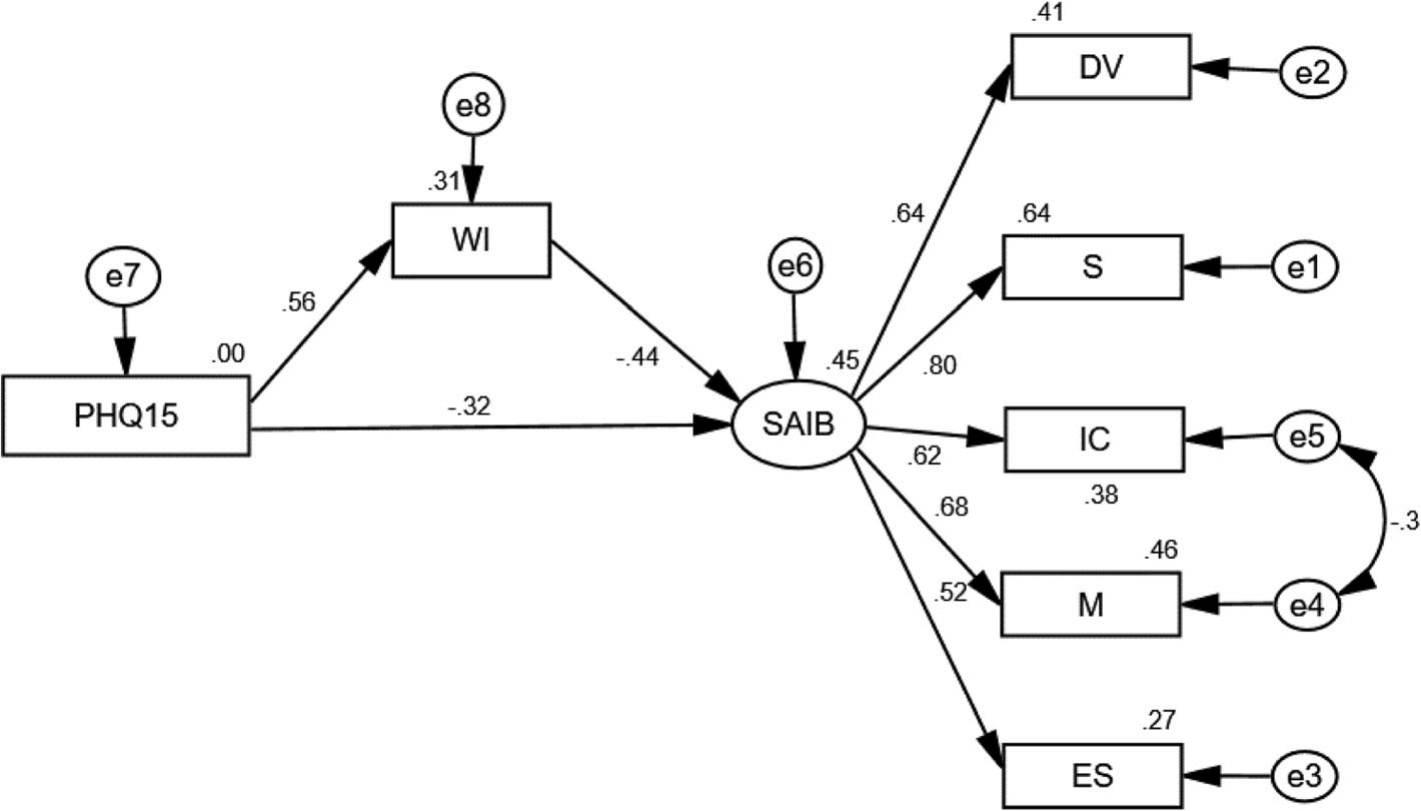
The complete mediation model (*N* = 323). Path coefficients were standardized. Note: WI-7: Whiteley Index-7 (assessing health anxiety); SAIB: the Scale for the Assessment of Illness Behavior (assessing illness behavior); PHQ-15: the Patient Health Questionnaire (assessing somatic symptoms);DV: Diagnosis evaluation; ES: expression symptoms; MT: medication and treatment; IC: Illness consequences; S: Scanning

SEM was used to explore the mediating role of HA in the relationship between FSSs and IB. Most of the fit indicators (CMIN/DF = 4.89, IFI = 0.93, NFI = 0.91, CFI = 0.93, SRMR = 0.05) were acceptable, except RMSEA = 0.11. Therefore, we modified the model according to modification indices, revealing correlation between error3 and error4, which were latent variables of IB. After updating the model, the mediation model (see Fig. 1) showed an acceptable fit (CMIN/DF = 3.79, IFI = 0.95, NFI = 0.94, CFI = 0.95, SRMR = 0.04, RMSEA = 0.09), and all path coefficients were significant at the 0.001 level. The significance of the mediating effects of HA was tested by the bootstrap estimation procedure in AMOS (a bootstrap sample of 5000 was specified). As displayed in Table 2, SSs exerted significant indirect effects on IB via HA. HA also had a partial mediating effect in the relationship between SSs and IB; the indirect effects accounted for 42.86% (Indirect effect/Total effect) of the total effect.

**Table 2:** Total effect, direct effect, indirect effect

	Path	β(standardization)	Bootstrop 95% CI	*P*
PHQ-15 → SAIB	Total effect (c)	−0.56	− 0.63~ − 0.48	< 0.001
	Direct effect (c’)	−0.32	− 0.43~ − 0.21	< 0.001
	Indirect effect (a x b)	−0.24	− 0.31~ − 0.18	< 0.001

Note: SAIB: the Scale for the Assessment of Illness Behavior (assessing illness behavior); PHQ-15: the Patient Health Questionnaire (assessing somatic symptoms)

### Moderate mediation model

The PHQ-15 score was set as an independent variable and SAIB total score as a dependent variable, the WI-7 score had a mediating effect, and age was a moderating variable. Multiple regression analysis was performed to test which path in the mediation model was adjusted. The result showed that the effect of age on the relationship between FSSs and HA was statistically significant. However, there was no statistically significant effect of the moderating in the relationship between HA and IB, and there was also no statistically significant moderating effect on the relationship between FSSs and IB (Table 3).

**Table 3:** Multiple regression analysis results with moderate mediation

	Dependent variable:WI				Dependent variable:SAIB			
	coef	SE	t	p	coef	SE	t	*p*
PHQ15	0.386	0.033	11.639	0.001				
AGE	0.002	0.001	2.432	0.016				
PHQ15*AGE	−0.008	0.003	−0.2836	0.005				
WI					−0.641	0.100	−.6.411	0.001
AGE					−0.002	0.002	−1.057	0.291
WI * AGE					−0.001	0.008	−0.078	0.938
PHQ15					−0.370	0.071	−5.252	0.001
AGE					−0.002	0.002	−1.057	0.291
PHQ15*AGE					−0.003	0.006	−0.474	0.636

Note: WI-7: Whiteley Index-7 (assessing health anxiety); SAIB: the Scale for the Assessment of Illness Behavior (assessing illness behavior); PHQ-15: the Patient Health Questionnaire (assessing somatic symptoms)

**Fig. 2:**
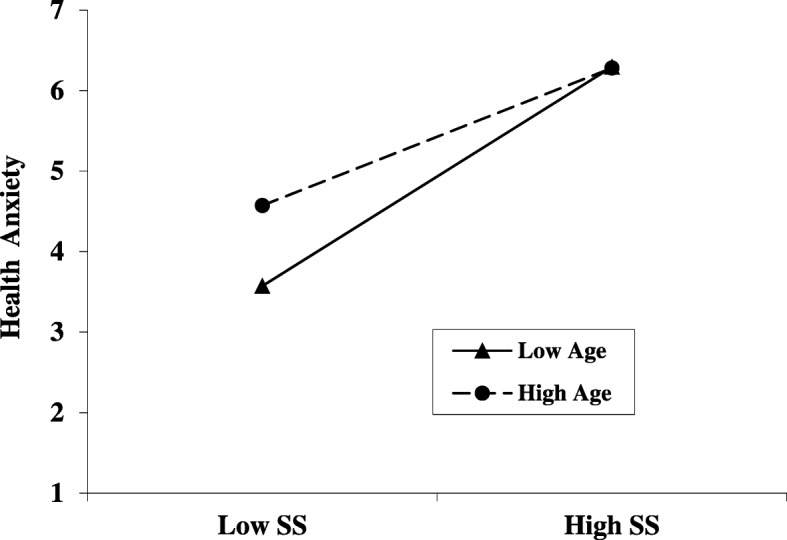
The moderating effect of age on the association between FSS and HA. Note: The ordinate is the total score of health anxiety, and the abscissa: low SS refers to somatic symptoms (M-SD), High SS refers to somatic symptoms (M + SD); Low Age: age (M-SD), High Age: age (M + SD)

To further test the moderating effects of age between SSs and HA, subjects who aged above M + SD (33.84 + 12.35) were set as the high age group and below M − SD (33.84–12.35) as the low age group. The results showed that the moderating effect was established in the low age group (effect = 0.48; 95% CI [0.39 ~ 0. 57]), and the moderating effect also existed in the high age group (effect = 0.29; 95% CI [0.20~0.38]). Furthermore, there was a significant difference between two age groups (t = − 2.7585; *p* = 0.0061). Those indicated a moderate–mediation model. (Fig. 2).

## Discussion

The present study examined the role of HA in the relationship between FSSs and IB using SEM. The main hypothesis was partly supported by the results; HA was shown to partially mediate the association between FSSs and IB in a sample of the inpatients with clinical depression. More specifically, severer FSSs increased more abnormal IB by bringing about higher HA. We also found that age significantly moderated the impact of FSSs on HA with older patients experiencing higher HA for mild FSSs.

### Partial mediation of health anxiety

Correlational analyses found an intensifying relationship between any two of FSS, HA, and IB. These findings are consistent with most previous literature suggesting that both elevated HA and severer SSs are significantly associated with more abnormal IB [9, 36–39] and with prior studies suggesting that severer SSs are linked to elevated HA [15, 17].

More importantly, according to the results of SEM, HA accounted for 42.86% of the total effect of FSSs on IB. In other words, increasing HA is an indirect approach of FSSs to elevate IB. To the best of our knowledge, this is the first study verifying the mediating role of HA between FSSs and IB and clarifying the portion of this mediating effect in the total effect of FSSs. Given that the remaining total effect may consist of a direct effect of FSSs and indirect effects of other variables beyond the present study’s interest, this finding emphasizes that HA is an important intermediate mechanism when we consider how FSSs influence IB.

The sample of the present study was inpatients with depression, most of them with FSSs, which should be noted to understand the results. It is well known that depression is often accompanied by FSSs [1, 3]. Depression and anxiety often accompany each other, and severe HA was suggested to be reclassified as an anxiety disorder in the DSM-V [40]. Hence, we speculate that patients with depression may have higher anxiety qualities than other people. These people are more sensitive and excessively attend to their own specific signs and symptoms, subsequently worrying about their health and attributing these FSSs to organic causes rather than functional ones, resulting in elevated HA. Typically, patients with high HA tend to hold the catastrophic interpretation with FSSs (this headache means a brain tumor) [41]. To seek reassurance about the fear of such symptoms and to alleviate this kind of HA, patients often enact inappropriate IB [36, 42]. Therefore, it seems reasonable that HA would be a potent mediating factor of this association between FSSs and IB.

### Age effect

Another interesting finding was that the age of patients with depression played a moderating role in the relationship between FSSs and HA. Although there were significant positive relationships between FSSs and HA in both of younger and older groups, a more thorough analysis revealed the age effect. Specifically, compared with the younger group, the older patients had higher HA when FSSs were mild. Given that both groups reached the same levels of HA when FSSs were severe, this pattern of age effect suggests that older patients with depression may overreact to mild FSS.

It is necessary to contrast the age effect found in the present study with the findings of previous studies. Previous research showed that older adults (over age 64) report relatively low levels of worrying about health issues than do college students [43]. The current research indicated that level of HA was higher in the high-aged group (33.84 + 12.35) than in the low-aged group (33.84–12.35), but this growth trend gradually slowed with increasing age. The explanation for the inconsistent result may be that our study focused on a depressed population who may have high anxiety characteristics while the prior research focused on the general population. Another hypothesis from other researchers is that HA may peak at a certain age and decrease thereafter [44, 45]. Our study has an age range from 14 to 68 years; therefore, it is necessary to investigate in a larger age range allowing for comparison across the entire lifespan.

### Implications for treatment

The results of the present study have multifaceted value for clinical practice with patients with depression disorder. First, the partially mediating role of HA between FSSs and IB highlights the need to pay more attention to HA (especially in high-aged patients with depression with SSs, even if mild) when treating depression disorder rather than dealing only with the depressive symptoms. Second, the results suggest that HA can be a promising intervention target for reducing depressive patients’ inappropriate IB. Therefore, we recommend a regular assessment of FSSs and HA in patients with depression. For those with mild HA, the doctor should explain the nature of the symptoms to the patient, thereby avoiding more unnecessary HA and IB. For those with severe HA, timely and effective intervention is necessary. In addition, it is recommended to actively evaluate and intervene in HA in elderly patients with depression with FSS (even if mild).

Currently, the effectiveness of antidepressants in treating FSSs is far from ideal; there are often residual physical symptoms [46, 47]. Studies have shown that the treatment response of HA was much better than FSSs [48]. Thus, the HA intervention becomes more important and efficient in reducing the IB of patients with depression. High levels of health anxiety might be a multidimensional trait, where triggering factor (such as physiological processes), cognitive and behavioral strengthen each other. Although avoiding triggers and using safe behaviors (such as more examinations of symptoms) may lead to a reduction in health anxiety in the short term but higher levels of health anxiety in the long run [49]. Fortunately, psychoeducation, exposure and response prevention, antidepressants, and cognitive restructuring techniques might be helpful for patients with severe health anxiety. Specifically, cognitive-behavioral therapy has been reported to be a highly effective treatment for hypochondriasis/HA [18, 50, 51], even better than drug treatment [52]. The strengths of the cognitive behavioral model of severe health anxiety lies in its account of maintaining factors. The maintenance factor of health anxiety lies in the faulty cognitive belief of bodily sensations and external events. Consequently, the hallmark of cognitive therapy is that it entails components aiming to change these faulty beliefs. It can alleviate HA by helping patients recognize and modify false beliefs about the symptoms [41] . A recent study found that cognitive-behavioral therapy can reduce perceived risk of disease, attention to bodily symptoms, and intolerance of uncertainty significantly to improve HA [53].

### Limitations

A number of limitations of the present study should be considered. First, given the cross-sectional design, the current study could not infer the causal nature of the associations among FSS, HA, and IB. Thus, future study should examine the associations of these variables using a longitudinal design or an experimental manipulation method that allows causal relationship. Second, the present study examined the mediating role of HA in the relationship between FSSs and IB in a clinical depressed sample, whether this conclusion can be extended to other mental disorders requires further confirmation. Therefore, future research should utilize a longitudinal design to examine this association in other samples to determine the clinical relevance of the findings. Third, we did not consider other factors that previous studies have reported that may affect IB, such as personality traits [54] and depression [7] . It is worth mentioning that we found no association between SAIB and the depression scale of Patients Health Questionnaire-9 in the present study, which is consistent with the finding of Rief et al. [29]. While this result is in contrast to results of Wilson Barnett and Trimble who using Illness Behavior Questionnaire (IBQ) as assessment tool [55]. The possible reason for this inconsistence may be that IBQ focus on evaluation of emotional aspects and hypochondriacal concerns rather than aspects of illness behavior (SAIB). However, because the subjects recruited are hospitalized patients with severe symptoms of depression in our study, depression may still have certain effects on variables in the mediation model. It would be helpful to validate the mediation model in the general population. Despite these limiting factors, the strength of this study is the emphasis on the mediating effect of health anxiety in clinical major depressed samples. In future, the result can be further validated in a population-based study and non-hospitalized sample in order to control these effects.

## Conclusion

In conclusion, the current study reports the partial mediating role of HA in the relationship between FSSs and IB in a Chinese sample of inpatients with depression, which was moderated by age. The results of the present study emphasize the importance of HA in patients with depression with SSs, and it is necessary to evaluate and intervene in HA appropriately, especially in older patients.

## Data Availability

The datasets used and/or analyzed during the current study are available from the corresponding author on reasonable requests.

## Abbreviations

FSS: functional somatic symptoms
HA: Health anxiety
IB: Illness behavior
IBQ: Illness Behavior Questionnaire
PHQ-15: Patient Health Questionnaire
SAIB: The scale for the Assessment of Illness Behavior
SS: Somatic symptoms
WI-7: The Whiteley Index-7
